# Risk Perception in a Real-World Situation (COVID-19): How It Changes From 18 to 87 Years Old

**DOI:** 10.3389/fpsyg.2021.646558

**Published:** 2021-03-02

**Authors:** Alessia Rosi, Floris Tijmen van Vugt, Serena Lecce, Irene Ceccato, Martine Vallarino, Filippo Rapisarda, Tomaso Vecchi, Elena Cavallini

**Affiliations:** ^1^Department of Brain and Behavioral Sciences, University of Pavia, Pavia, Italy; ^2^Department of Psychology, University of Montreal, Montreal, QC, Canada; ^3^Department of Neuroscience, Imaging and Clinical Sciences, University G. d’Annunzio of Chieti-Pescara, Chieti, Italy; ^4^Sociosfera ONLUS SCS, Seregno, Italy; ^5^Cognitive Psychology Unit, IRCCS Mondino Foundation, Pavia, Italy

**Keywords:** risk perception, COVID-19, anxiety, emotion, availability heuristic

## Abstract

Studies on age-related differences in risk perception in a real-world situation, such as the recent COVID-19 outbreak, showed that the risk perception of getting COVID-19 tends to decrease as age increases. This finding raised the question on what factors could explain risk perception in older adults. The present study examined age-related differences in risk perception in the early stages of COVID-19 lockdown, analyzing variables that can explain the differences in perception of risk at different ages. A total of 1,765 adults aged between 18 and 87 years old completed an online survey assessing perceived risk severity and risk vulnerability of getting COVID-19, sociodemographic status, emotional state, experience relating to COVID-19, and physical health status. Results showed that the older the participants, the lower the perceived vulnerability to getting COVID-19, but the higher the perceived severity. Different predictors explain the perception of risk severity and vulnerability at different ages. Overall, self-reported anxiety over the pandemic is a crucial predictor in explaining risk perceptions in all age groups. Theoretical and practical implications of the empirical findings are discussed.

## Introduction

Negative events are a part of life, and the likelihood or severity we ascribe to them affects how we act and feel in relation to these events. It is therefore important to understand what governs risk perception, especially so in the case of emerging realities such as the recent COVID-19 outbreak.

Risk perception is a cognitive process that guides people’s behaviors in the face of situations involving potential risks. Two models in the field of health, the Protection Motivation Theory (PMT; Rogers, 1983) and the Health Belief Model (HBM; Becker, 1974), have proposed a distinction between two different aspects of risk perception: the subjective probability of contracting a health condition (perceived vulnerability), and the degree to which we are concerned (perceived severity) about its consequences.

Few studies have examined the age-related differences in risk perception using hypothetical real-life situations (e.g., Hanoch et al., 2018; Sun and Sun, 2019). Recently, the COVID-19 outbreak gave researchers the opportunity to focus on the perception of risk across the lifespan applied to a real-life situation (Bruine de Bruin, 2020; Pasion et al., 2020; Guastafierro et al., 2021; Kivi et al., 2021). These studies revealed that the perceived vulnerability to COVID-19 tends to decrease as age increases. However, older adults perceived higher risk of dying because of COVID-19 (Bruine de Bruin, 2020). These important findings raised the question of what factors could explain risk perception in older adults. The HBM (Becker, 1974) showed that individual beliefs about risks can be influenced by different modifying factors, such as sociodemographic and sociopsychological variables, and knowledge/experience.

Concerning sociodemographic factors, gender, education, and employment are believed to have an influence on risk perception. Indeed, recent studies on COVID-19 found that these variables predicted both risk perception (Bruine de Bruin, 2020; Dryhurst et al., 2020; Yıldırım and Güler, 2020) and preventive/protective behaviors during pandemics (e.g., Carlucci et al., 2020; Li et al., 2020; Roma et al., 2020). Regarding sociopsychological factors, one variable that is likely to influence risk perception and decision-making behavior is the emotional state (Loewenstein et al., 2001). People in a positive emotional state tend to evaluate events more favorably than participants in a negative emotional state (Slovic and Peters, 2006). Analyzing the relationship between emotion and risk perception is relevant particularly in older adults because changes in emotional experience are observed in this population. Indeed, the socio-emotional selectivity theory (SST; Carstensen et al., 1999) posits that older adults attribute more importance to emotions than to knowledge acquisition, respect to younger people, and this shift makes the emotional state a potential predictor of their risk perception. Furthermore, anxiety can affect risk perception, since it helps individuals deal with dangerous environments presenting potential threats (Barlow, 2002). Individuals with high levels of anxiety tend to perceive negative outcomes as more likely (higher vulnerability) and severe (Stöber, 1997). Risk perception also depends on knowledge/experiences that form the basis on which people estimate risk. Indeed, the frequency of an event and the ease with which it can be recalled or imagined influence people’s risk perception (Tversky and Kahneman, 1982). The availability heuristic suggests that people who have a recent personal experience of a specific event have higher risk perception (Slovic et al., 2004). Recent studies found that the perceived vulnerability to COVID-19 was higher in individuals who knew someone infected with it (Liu et al., 2020; Guastafierro et al., 2021), presumably because they used this experience as a cue (Peters et al., 2006).

The present study aimed to assess age differences in risk perception in a real-life situation, the COVID-19 pandemic, and to understand which variables, such as sociopsychological and sociodemographic variables and knowledge/experience, can explain the perception of risk at different ages. Participants were invited to complete an online survey conducted during the early stages of COVID-19 lockdown in Italy, assessing their perception of risk severity and risk vulnerability, sociodemographic status, emotional state, experiences with COVID-19 and health status.

We expected to find age-related differences in risk perception (H1) and a different pattern of predictors of risk perception in younger vs. older adults (H2). Specifically, we hypothesized that older adults would perceive higher risk severity (Bruine de Bruin, 2020) but less vulnerability risk of getting COVID-19 than younger adults (Bruine de Bruin, 2020; Pasion et al., 2020; Guastafierro et al., 2021; Kivi et al., 2021). Moreover, in line with the SST (Carstensen et al., 1999), we expected that older people experienced fewer negative emotions and less anxiety, and that their risk perception was determined more by emotions compared to younger adults. However, since older adults are better than younger adults in regulating their emotions (Carstensen et al., 1999) a possible alternative hypothesis is that the emotional state loses importance in predicting risk perception as individuals age. Moreover, since older adults are generally found to be less interested in knowledge acquisition than younger adults and show a decline in cognitive functioning (Salthouse, 2019), we expected that the evaluation of the impact of COVID-19 on the local community (relatives/friends/acquaintances) would not be critical predictor of risk perception in older people. This was expected because the estimation of the impact would require additional cognitive resources. Because it was previously shown that anxiety (Stöber, 1997), physical health status, and sociodemographic factors (e.g., Bruine de Bruin, 2020; Dryhurst et al., 2020) predict risk perception, we expected them to be significant predictors of risk perception regardless of age.

## Methods

### Participants

The study was conducted through an anonymous online survey among adult Italian residents between April 9th and May 3rd, 2020 – the earlier stages of COVID-19 outbreak in Italy. Participants were a convenience sample selected on the basis of their accessibility to the online survey. Inclusion criteria were: (a) age 18 years or older, (b) place of residence in Italy during the compilation of the survey, and (c) signing the informed consent.

Out of 2,625 respondents who accessed the online survey, a total of 1,765 completed the questionnaire (response rate = 67.24%). Participants’ age ranged from 18 to 87 (*M* = 47.14; *SD* = 15.93). We divided the sample in six continuous age groups: age range 18–29 (*n* = 288), age range 30–39 (*n* = 374), age range 40–49 (*n* = 306), age range 50–59 (*n* = 318), age range 60–69 (*n* = 324), over 70 (*n* = 155). Demographic characteristics are summarized in Table 1. The 40–49 and 50–59 were those age groups reported to work in a hospital setting (10.5 and 10.7%, respectively), and, among respondents, 0.8% reported having been diagnosed with COVID-19.

**TABLE 1:** Socio-demographic characteristics by age groups.

	Age groups							
*Characteristics* (*n*,%)	18–29 (*n* = 288)	30–39 (*n* = 374)	40–49 (*n* = 306)	50–59 (*n* = 318)	60–69 (*n* = 324)	Over 70 (*n* = 155)	*X*^2^ or *F (df)*	*p*
**Age** (*M; DS*)	24.83 (3.14)	34.29 (2.86)	44.52 (2.80)	54.54 (2.85)	63.96 (2.79)	74.45 (3.85)	9705.84	<0.001
**Gender**							50.07 (10)	<0.001
Female	219 (76)	283 (75.7)	225 (73.5)	254 (79.9)	232 (71.6)	82 (52.9)		
Male	69 (24)	90 (24.1)	81 (26.5)	62 (19.5)	92 (28.4)	73 (47.1)		
Not specified	0 (0)	1 (0.3)	0 (0)	2 (0.6)	0 (0)	0 (0)		
**Education**							83.46 (5)	<0.001
Not having university degree	120 (41.7)	134 (35.8)	169 (55.2)	204 (64.2)	199 (61.4)	85 (54.8)		
Having university degree	168 (58.3)	240 (64.2)	137 (44.8)	114 (35.8)	125 (38.6)	70 (45.2)		
**Marital status**							257.85 (5)	<0.001
Unmarried	228 (79.2)	118 (31.6)	99 (32.4)	97 (30.5)	79 (24.4)	44 (28.4)		
Married	60 (20.8)	256 (68.4)	207 (67.6)	221 (69.5)	245 (75.6)	111 (71.6)		
**Employment**							516.52 (5)	<0.001
Not working	139 (48.3)	26 (7)	15 (4.9)	38 (11.9)	165 (50.9)	117 (75.5)		
Working	149 (51.7)	348 (93)	291 (95.1)	280 (88.1)	159 (49.1)	38 (24.5)		
**Region of residence**							23.78 (10)	0.008
Northern Italy	268 (93.1)	356 (95.2)	295 (96.4)	300 (94.3)	297 (91.7)	142 (91.6)		
Center Italy	10 (3.5)	6 (1.6)	7 (2.3)	11 (3.5)	19 (5.9)	18.5 (7.7)		
Southern Italy	10 (3.5)	12 (3.2)	4 (1.3)	2 (2.2)	8 (2.5)	1 (0.6)		
**Working in hospital setting**	10 (3.5)	17 (4.5)	32 (10.5)	34 (10.7)	14 (4.3)	1 (0.6)	36.63 (5)	<0.001
**Diagnosis with COVID-19**	2 (0.7)	0 (0)	6 (2)	4 (1.3)	2 (0.6)	0 (0)	10.57 (5)	0.061

*For the variable age, the table reports means and (deviation standard). For all other characteristics, the table reports the frequency and (percentage).*

### Procedure

The questionnaire was administered cross-sectionally through a web-based survey on LimeSurvey^®^ which participants accessed via a designated link. The link was distributed via e-mail and social network messaging. We used a snowball sampling technique, asking participants to share the survey link to others within their network. The survey took approximately 15 min to complete and participants did not receive financial compensation. All participants provided their consent to participate.

### Measures

#### Risk Perception

We developed a questionnaire based on prior studies on risk perception during the previous pandemic influenza (Bults et al., 2011) in order to measure (Table 2): (a) risk severity (three items), i.e., how serious contracting the virus would be for a person’s health, and (b) risk vulnerability (three items), i.e., a person’s perception of the risk to contract the virus. Responses were provided on a 5-point Likert scale (1 = *not at all*; 5 = *extremely*). We created an index of risk severity (Cronbach’s alpha = 0.81) and risk vulnerability (Cronbach’s alpha = 0.75), respectively, by summing the scores of items.

**TABLE 2:** Questions of the online questionnaire about risk perceptions (risk severity and risk vulnerability), perceived anxiety about COVID-19, previous experience of COVID-19 and health perception.

Questions
**Risk severity^**§**^**
How severe do you think COVID-19 is?
How awful it would be if you were to be diagnosed with COVID-19 in the next 12 months?
How harmful is COVID-19 for my health?
**Risk vulnerability^**§**^**
In general, how much do you think you are susceptible to getting COVID-19?
How likely is it that you will be diagnosed with COVID-19 in the next 12 months?
How likely is it that you will be diagnosed with COVID-19 in the next 12 months, compared to others of your sex and age in Italy?
**Perceived anxiety about COVID-19^**
Are you worried about COVID-19?
Are you scared to get COVID-19?
How often do you think about COVID-19 in a day?
Are you worried that a relative of yours would be affected by COVID-19?
Are you worried that a friend of yours would be affected by COVID-19?
**Experience with COVID-19**
How many people you know (relatives, friends, acquaintance) have been infected with COVID-19?
How many people you know (relatives, friends, acquaintance) have died due to COVID-19?
**Objective health**
Do you have previous pathologies?
**Subjective health**
How is your health compared to the average of people of your age?

*^§^We used the same items used by Bults et al. (2011) to measure the perception of risk severity and risk vulnerability. ^The first three questions are taken from Bults et al. (2011), while the last two questions were developed for the present study.*

#### Sociodemographic Variables

We assessed socio-demographic variables by asking participants’ gender (male = 0; female = 1); education (not having university degree = 0; having university degree = 1), marital status (not married = 0; married = 1); and employment (not working = 0; working = 1).

#### Emotional State During the COVID-19 Emergency

We used the 37-item version of the Profile of Mood States (POMS; Shacham, 1983) in order to measure participants’ emotional state. People are requested to indicate how much they felt an emotion on a 5-point Likert scale (0 = *not at all*; 4 = *extremely*), during the last week. Six scores are derived: Tension-Anxiety (six items; e.g., anxious, nervous), Depression-Dejection (eight items; e.g., unhappy, sad), Anger-Hostility (seven items; e.g., angry, annoyed), Vigor-Activity (six items; e.g., energetic, active), Fatigue-Inertia (five items; e.g., exhausted, weary), and Confusion-Bewilderment (five items; e.g., confused, bewildered). For each scale, a total score was computed by averaging items’ responses.

In addition, questions probing feelings of anxiety about COVID-19 using measures of anxiety adapted from Bults et al. (2011) were also added (Table 2). Responses were provided on a 5-point Likert scale (1 = *not at all*; 5 = *extremely*). A total score was computed by averaging items’ responses (Cronbach’s alpha = 0.81).

#### Experiences With COVID-19

We assessed real-life experience with COVID-19 by asking participants the number of known (diagnosed) COVID-19 cases that have been infected and/or died among their relatives/friends/acquaintances (Table 2). For each question participants could respond the following: nobody, 1–5 people, 6–15 people, or more than 15 people.

#### Objective and Subjective Physical Health

Objective physical health status was assessed by asking participants to indicate if they had any health problems among a list of five chronic conditions (immunodepression, respiratory disease, heart disease, diabetes, and cancer) that are medically associated with increased severity of COVID-19 (Sanyaolu et al., 2020; Table 2). Subjective physical health status was measured by asking participants to rate their health (0 = *low*; 4 = *excellent*) compared to other people of their age (Table 2).

### Statistical Analyses

First, to assess the relationships between risk perception, age, and the other variables included in the study, we computed a correlation analysis between the risk perceptions and age, and sociodemographic variables, emotional states, experience with COVID-19, and physical health status.

Subsequently, in order to investigate age-related differences, we conducted a MANCOVA on perceptions of risk severity and risk vulnerability as dependent variables, and age groups (18–29 vs. 30–39 vs. 40–49 vs. 50–59 vs. 60–69 vs. over 70) as independent variables, entering gender, education, marital status, and employment as covariates. *Post hoc* pairwise comparisons were performed with Bonferroni’s correction for multiple comparison at *p* < 0.05.

Finally, in order to investigate which variables were most relevant for predicting risk perception, we conducted, separately for each age group, hierarchical regression analyses on risk severity and risk vulnerability as dependent variables. In these regressions we entered variables that were significantly associated with risk severity and risk vulnerability in correlation analyses. We entered into the models, sociodemographic variables at Step 1, emotional states at Step 2, risk severity/vulnerability at Step 3, experiences with COVID-19 at Step 4, and physical health status at Step 5. Variables were added in this order based on prior literature of the hypothesized relative importance (see section “Introduction”).

## Results

### Relationship Between Age and Risk Perceptions, and Sociodemographic Variables, Emotional States, Experience With COVID-19, and Physical Health Status

Age was positively associated with perceived severity (*p* < 0.001), and negatively with perceived vulnerability (*p* < 0.001). See Supplementary Table 1. Older adult age was correlated with gender (*p* < 0.001, higher age was associated with female), education (*p* < 0.001, higher age was associated with not university degree), marital status (*p* < 0.011, higher age was associated with being married), and employment (*p* < 0.001, higher age was associated with not working). Regarding emotional state, relatively older adults scored lower on all POMS subscales (*ps* < 0.001) with the exclusion of the vigor-activity subscale that was positively associated with age (*p* = 0.025). Perceived anxiety for COVID-19 was unrelated to age (*p* = 0.129). Relatively older adults reported a low incidence of COVID-19 among relatives/friends/acquaintances (*p* = 0.013), having more health chronic conditions (*p* < 0.001), and a lower subjective physical health (*p* = 0.003).

Perceived severity and perceived vulnerability were significantly correlated (*p* < 0.001). Perceived severity was correlated with gender (*p* < 0.001, higher severity was associated with female), education (*p* < 0.001, higher severity was associated with not having university degree), marital status (*p* < 0.001, higher severity was associated with being married), and employment (*p* = 0.046, higher severity was associated with not working). It was associated with some emotional states: positively with perceived anxiety about COVID-19 (*p* < 0.001) and the POMS subscale tension-anxiety (*p* = 0.033), negatively with the subscale anger-hostility (*p* = 0.042). Moreover, perceived severity was positively associated with objective physical health (*p* < 0.001) and the incidence of deaths among relatives/friends/acquaintances (*p* < 0.001), and negatively associated with subjective physical health (*p* < 0.001).

Perceived vulnerability was correlated with gender (*p* < 0.001, higher vulnerability was associated with female), education (*p* = 0.034, higher vulnerability was associated with having university degree) and employment (*p* < 0.001; higher vulnerability was associated with working). It was positively associated with all emotional states (both perceived anxiety about COVID-19, *p* < 0.001, and all POMS subscales, *ps* < 0.020), as well as with the incidence of infections (*p* < 0.001) and deaths (*p* < 0.001) among relatives/friends/acquaintances, and negatively associated with subjective physical health (*p* < 0.001).

### Age Differences in Risk Perceptions

**FIGURE 1:**
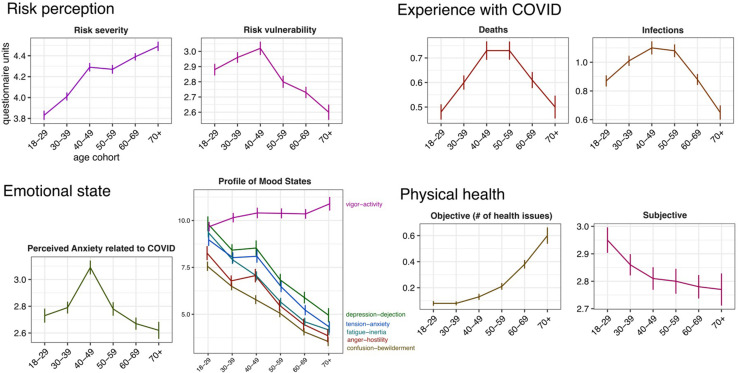
Age-related differences in risk perception, emotional state, experience with COVID-19, and physical health. The horizontal axes indicate the age cohorts. Units on the vertical axes correspond to standard units of the questionnaires used (see text for details). Error bars represent standard error of the mean. For emotional state, experience with COVID-19 and physical health, age cohorts were computed for presentation purposes only.

MANCOVA showed age-group differences in risk severity, *F*(5,1755) = 24.54, *p* = 0.001, and risk vulnerability, *F*(5,1755) = 6.25, *p* < 0.001, controlling for sociodemographic variables. Analyses indicated that adults over 70 years perceived higher risk severity of COVID-19 compared to 18–29, 30–39, 40–49, and 50–59 (*ps* < 0.024; see Figure 1; Supplementary Table 2). Conversely, younger adults 18–29 and 30–39 perceived lower risk severity of COVID-19 compared to all other age groups (*ps* < 0.006). Regarding the risk vulnerability of COVID-19, older adults over 70 reported lower risk compared to younger age groups 18–29, 30–39, 40–49 (*ps* < 0.026). Age groups 50–59 and 60–69 reported lower risk vulnerability compared to 40–49 (*ps* = 0.002).

### Effect of Sociodemographic Variables, Emotional States, Experience With COVID-19, and Physical Health Status on Risk Perceptions in Each Age Groups

Regarding risk severity (see Supplementary Tables 3,4), the hierarchical regression analysis showed that in age group 18–29, Step 2 explained 39% of the variance, where perceived anxiety about COVID-19 was the only predictor. In age groups 30–39 and 40–49, Step 1 explained 8% and 9% of the variance, respectively, where gender and education were predictors. The addition in Step 2 of perceived anxiety about COVID-19 – and only for age group 40–49 also POMS subscale anger-hostility – led to an increase of 28% in variance explained. However, in the last step education was no longer a predictor. In age groups 50–59 and over 70, Step 2 explained 36% and 15% of the variance, respectively, where perceived anxiety about COVID-19 and the POMS subscale anger-hostility were predictors. In age group 60–69, Step 1 explained 3% of the variance, where employment was a predictor. The addition in Step 2 of perceived anxiety about COVID-19 and in Step 3 of risk vulnerability led to an increase of 25% and 10% in variance explained, respectively. However, in the last step risk vulnerability was no longer a predictor.

For risk vulnerability (see Supplementary Tables 5, 6), in the age group 18–29, Step 2 explained 19% of the variance, with the perceived anxiety about COVID-19 being a predictor. The addition in Step 4 of experience of COVID-19 infection and in Step 5 of subjective physical health, led to an increase of 4% and 1% in variance explained. In age group 30–39, Step 1 explained 2% of the variance where gender and employment were predictors. The addition in Step 2 of the POMS subscale tension-anxiety and perceived anxiety about COVID-19, and in Step 4 the addition of experience of COVID-19 infection, led to a significant increase of 12% and 2% in variance explained, respectively. However, in the last step gender was no longer a predictor. In age group 40–49, Step 1 explained 4% of the variance where gender was a predictor. The addition in Step 2 of the perceived anxiety about COVID-19, and in Step 4 of experience of COVID-19 infection, led to an increase of 14% and 5% in variance explained. For the age group 50–59, Step 1 explained 3% of the variance where employment was a predictor. The addition in Step 2 of the perceived anxiety about COVID-19 and the POMS subscale anger-hostility, and in Step 3 of risk severity, in Step 4 of experience of COVID-19 infection, and in Step 5 of subjective physical health, led to an increase of 12%, 1%, 3%, and 2% in variance explained, respectively. However, in the last step employment was no longer a predictor. For the age group 60–69, Step 2 explained 15% of the variance where perceived anxiety about COVID-19 was the predictor. The addition in Step 4 of experience of COVID-19 infection led to an increase of 3% in variance explained. Finally, in the age group over 70, Step 2 explained 21% of the variance, with the perceived anxiety about COVID-19 being the only predictor. In Table 3 a schema of predictors of risk vulnerability and risk severity in the different age groups is reported.

**TABLE 3:** Summary of the significant predictors (identified with the X in the table) of risk severity and risk vulnerability at different ages.

	Age groups					
	18–29 (*n* = 288)	30–39 (*n* = 374)	40–49 (*n* = 306)	50–59 (*n* = 318)	60–69 (*n* = 324)	Over 70 (*n* = 155)
**Risk severity**						
Perceived anxiety	X	X	X	X	X	X
POMS anger-hostility			X	X		X
Gender		X	X			
Employment					X	
**Risk vulnerability**						
Perceived anxiety	X	X	X	X	X	X
Incidence infections	X	X	X	X	X	
Subjective health	X			X		
POMS anger-hostility				X		
POMS tension-anxiety		X				
Gender			X			
Employment		X				

## Discussion

The present study examined age-related differences in risk perception in a real-life situation, namely the early phase of the COVID-19 outbreak in Italy, analyzing variables that can explain the perception of risk at different ages from 18 to 87 years old.

We found that the perception of risk severity increased with age from young to older adult groups. Conversely, the perception of risk vulnerability was higher in younger than older adult groups. Our findings are consistent with those of recent research on COVID-19 (Bruine de Bruin, 2020; Pasion et al., 2020; Guastafierro et al., 2021; Kivi et al., 2021). This pattern of results reveals that older adults have a different perception of risk vulnerability and risk severity and confirms the distinction between the two aspects of risk perception proposed by both the HBM (Rogers, 1983) and the PMT (Becker, 1974). Age-related differences in risk perception were present even controlling for sociodemographic variables suggesting that they cannot fully explain the age changes in risk perception.

Furthermore, in line with previous studies on COVID-19 (e.g., Bruine de Bruin, 2020; Carstensen et al., 2020; Ceccato et al., 2020), negative emotional states tended to decrease, and the positive ones tended to rise as age increases. Although the risk of severe illness from COVID-19 increases with age (Zhou et al., 2020), relatively older adults may be more able than younger ones to regulate their negative emotions and feeling of anxiety, focusing on positive emotions and engaging activities. Our findings support the view that in spite of the stressors elicited by the pandemic that threatens their health and well-being, older people display emotional resilience (Carstensen et al., 2020; Pearman et al., 2021).

Regarding the experience with COVID-19, age was associated with fewer cases of family/relatives/acquaintances infected by COVID-19, whereas no relationship was found with the number of COVID-19 deaths. This unexpected result may be due to a decline, on the part of the older adults, in cognitive resources or a suppression of negative events, that led respondents to underestimate the actual number of people infected or died. On the other hand, it may be that the reduced social network of older adults during the COVID-19 lockdown, have limited their knowledge about incidence of people affected by the virus.

Looking at correlation analyses, in line with the HBM (Rogers, 1983), we found that perceived vulnerability and severity were associated with sociodemographic, sociopsychological, and knowledge/experience variables. Crucially, we found that different sets of these variables explain risk perception at different ages.

As expected, the feeling of anxiety about COVID-19 was a significant constant predictor explaining the perception of risk severity in all age groups. People with higher levels of anxiety specific to COVID-19 reported higher levels of risk severity toward the virus. Regarding emotions, anger-hostility was a predictor of risk severity in adulthood (40–59) and over 70, indicating that participants with higher levels of anger-hostility reported lower risk severity. This is in line with studies suggesting that anger attenuates risk estimation (Lerner and Keltner, 2000). Moreover, gender was a predictor in age groups 30–39 and 40–49 showing that females reported higher levels of risk severity for COVID-19 than males. In addition, in line with recent studies (Bruine de Bruin, 2020; Yıldırım and Güler, 2020), not working predicted higher levels of risk severity in the age group 60–69.

Regarding the perception of risk vulnerability, anxiety about COVID-19 was a positive predictor in all age groups. Furthermore, experience with COVID-19 infections among relatives/friends/acquaintances was another predictor in explaining the level of risk vulnerability in all age groups, with the exception of people over 70. This result suggests that, in line with the availability heuristic (Tversky and Kahneman, 1982), individuals from 18 to 69 years old may have used their personal experience with COVID-19 as a cue to estimate their risk perception about the virus (Liu et al., 2020; Guastafierro et al., 2021). Participants over 70 years did not use heuristic to perceive the risk of COVID-19, possibly because of a reduction in cognitive resources. Looking at sociodemographic variables, in line with previous studies (Dryhurst et al., 2020; Yıldırım and Güler, 2020), only in age group 40–49, being female predicted high levels of risk vulnerability. In addition, in age group 30–39, having an employment predicted high levels of risk vulnerability (Bruine de Bruin, 2020). Looking at the other predictors, subjective health perception was a negative predictor in explaining risk vulnerability in the age groups 18–29 and 50–59. It may be that these age groups believe that having low physical health status could make them more prone to COVID-19. Regarding emotions, only feelings of tension-anxiety and anger-hostility predicted risk vulnerability, respectively, in the age groups 30–39 and 50–59. Younger adults aged 30–39 with higher levels of tension-anxiety reported lower levels of risk vulnerability. To note that this POMS subscale assesses levels of anxiety and tension in general and not specific anxiety for COVID-19. Instead, adults 50–59 years old with higher levels of anger-hostility reported lower risk vulnerability (Lerner and Keltner, 2000). Overall, self-reported COVID-anxiety seems to be a crucial predictor of risk perceptions in all age groups.

Overall, in adults from 18 to 69 years several variables explained risk perceptions, particularly for risk vulnerability. By contrast, in older adults over 70 risk perception is predicted only by anxiety about COVID-19, for both risk severity and vulnerability, and by anger-hostility for risk severity. These results are in line with the SST (Carstensen et al., 1999) showing that in an older population, emotions are more relevant than knowledge acquisition. We can also speculate that a decline in cognitive functioning may have reduced the relevance of previous experiences in explaining risk perception in older people (Salthouse, 2019). This is a critical point, as it can have negative consequences on limiting the older adults’ protective behaviors.

Our results are specific to risk perceptions in the health domain, and specifically to COVID-19, and thus may not be generalizable to other domains. Moreover, we recruited a convenience sample which is not necessarily representative of the entire population and the cross-sectional nature of our study does not allow the observation of changes of risk perceptions of COVID-19 over time across age groups. Finally, given the self-reported nature of the survey, participants’ responses may have been partially distorted by social desirability bias.

In conclusion, we corroborated previous findings that the perception of risk severity tends to increase with age, while the perception of risk vulnerability of getting COVID-19 tends to decrease gradually (Bruine de Bruin, 2020; Pasion et al., 2020; Guastafierro et al., 2021; Kivi et al., 2021). Thus, risk perception in a real-life situation does not show an overall increase with age, but vulnerability and severity show an opposite pattern. The present research has also practical implications, since the PMT (Rogers, 1983) postulates that individuals’ perceptions of the severity of and their vulnerability to the threat tend to inhibit maladaptive behaviors. Hence, lower levels of risk vulnerability reported by relatively older adults may lead them to underestimate risks associated to COVID-19, reducing protective responses toward the virus, as found in previous COVID-19 studies (Carlucci et al., 2020; Pasion et al., 2020; Roma et al., 2020).

Knowing the predictors of risk perception could help protecting older adults’ lives and establishing effective preventive actions. Indeed, since in older adults only anxiety about COVID-19 has predicted risk vulnerability, risk communication strategies should be designed by public health policymakers in order to improve protective behaviors in such population. This could be very useful for the ongoing and also for future emergencies.

## Data Availability Statement

The raw data supporting the conclusions of this article will be made available by the authors, without undue reservation.

## Ethics Statement

The studies involving human participants were reviewed and approved by Ethical Committee of the Department of Brain and Behavioral Sciences of the University of Pavia (n. 46/2020). The participants provided their written informed consent to participate in this study.

## Author Contributions

AR, EC, SL, and FTVV elaborated the design of the study and had major roles in writing the manuscript. AR was responsible for carrying out the statistical analysis. IC, MV, and FR elaborated the design of the study and assisted with writing the article. TV, EC, and SL had major roles in revising the article critically for intellectual content. All authors contributed to the article and approved the submitted version.

## Conflict of Interest

The authors declare that the research was conducted in the absence of any commercial or financial relationships that could be construed as a potential conflict of interest.
