# Supplementary material for: Risk Perception in a Real-World Situation (COVID-19): How It Changes From 18 to 87 Years Old
Source: Front Psychol. 2021 Mar 2;12:646558. doi: 10.3389/fpsyg.2021.646558 (PMC7961080; doi:10.3389/fpsyg.2021.646558)
Supplement: Supplementary file 1 [file Table_1.DOCX]

Supplementary Material

**Table S1.** *Pearson correlations between risk perceptions and age, and sociodemographic variables, emotional states, experiences with Covid-19, and perceived health in the whole sample.*

|  | **Risk severity** | **Risk vulnerability** | **Age** |
| --- | --- | --- | --- |
| Risk severity | - | 0.19*** | 0.28*** |
| Risk vulnerability | 0.19*** | - | -0.14*** |
| Age | 0.28*** | -0.14*** | - |
| Gender^§^ | 0.08*** | 0.11*** | -0.09*** |
| Education^§^ | -0.18*** | 0.05* | -0.17*** |
| Marital status^§^ | 0.11*** | 0.01 | 0.28*** |
| Employment^§^ | -0.06* | 0.14*** | -0.20*** |
| POMS depression-dejection | 0.01 | 0.16*** | -0.23*** |
| POMS confusion-bewilderment | 0.01 | 0.16** | -0.31*** |
| POMS tension-anxiety | 0.05* | 0.19*** | -0.27*** |
| POMS anger-hostility | -0.05* | 0.13*** | -0.24*** |
| POMS fatigue-inertia | -0.04 | 0.16*** | -0.39*** |
| POMS vigor-activity | -0.04 | -0.05* | 0.05* |
| Perceived anxiety | 0.54*** | 0.37*** | -0.04 |
| Incidence infections | 0.02 | 0.24*** | -0.06* |
| Incidence deaths | 0.11*** | 0.13*** | 0.04 |
| Objective health | 0.13*** | -0.03 | 0.30*** |
| Subjective health | -0.14*** | -0.13*** | -0.07** |
| Note. POMS = Profile of Mood State  ^***^ *p* < .001; ^**^ *p* < .01; ^*^ *p* < .05;  ^§^Spearman’s rho (r_s_) is reported.  Gender was coded 0 = male, 1 = female; Education was coded 0 = not having university degree, 1 = having university degree; Marital status was coded 0 = unmarried, 1 = married; Employment was coded 0 = not working; 1 = working. | | | |

**Table S2**. *Bonferroni post-hoc pairwise comparisons on age group differences in risk severity and risk vulnerability, when entering gender, education, marital status, and occupation status as covariates.*

|  | **Risk severity** | | **Risk vulnerability** | |
| --- | --- | --- | --- | --- |
|  | ***p-value*** | **95% CI** | ***p-value*** | **95% CI** |
| **18-29 vs. 30-39** | .106 | -0.34, -0.15 | 1.00 | -0.17, 0.17 |
| **18-29 vs. 40-49** | < .001 | -0.60, -0.23 | 1.00 | -0.25, .12 |
| **18-29 vs. 50-59** | < .001 | -0.54, -0.17 | .236 | -0.03, 0.33 |
| **18-29 vs. 60-69** | < .001 | -0.67, -0.32 | .110 | -0.01, 0.33 |
| **18-29 vs. over 70** | < .001 | -0.87, -0.44 | .023 | 0.02, 0.44 |
|  |  |  |  |  |
| **30-39 vs. 40-49** | < .001 | -0.41, -0.09 | 1.00 | -0.22, 0.09 |
| **30-39 vs. 50-59** | .007 | -0.35, -0.03 | .088 | -0.01, 0.30 |
| **30-39 vs. 60-69** | < .001 | -0.50, -0.17 | .071 | -0.01, 0.32 |
| **30-39 vs. over 70** | < .001 | -0.71, -0.28 | .026 | 0.15, 0.44 |
|  |  |  |  |  |
| **40-49 vs. 50-59** | 1.00 | -0.10, 0.23 | .002 | 0.05, 0.37 |
| **40-49 vs. 60-69** | 1.00 | -0.26, 0.09 | .002 | 0.05, 0.39 |
| **40-49 vs. over 70** | .024 | -0.47, -0.02 | .001 | 0.07, 0.51 |
|  |  |  |  |  |
| **50-59 vs. 60-69** | .208 | -0.23, 0.10 | 1.00 | -0.16, 0.18 |
| **50-59 vs. over 70** | .001 | -0.52, -0.08 | 1.00 | -0.13, 0.30 |
|  |  |  |  |  |
| **60-69 vs. over 70** | .330 | -0.04, 0.36 | 1.00 | -0.13, 0.27 |

**Table S3.** *Hierarchical regression analysis to predict risk severity in age groups 18-29, 30-39, and 40-49.*

|  | **Age group 18-29** | | | | **Age group 30-39** | | | | **Age group 40-49** | | | |
| --- | --- | --- | --- | --- | --- | --- | --- | --- | --- | --- | --- | --- |
| **Step and predictor variable** | ***B*** | ***SE B*** | ***β*** | ***ΔR^2^*** | ***B*** | ***SE B*** | ***β*** | ***ΔR^2^*** | ***B*** | ***SE B*** | ***β*** | ***ΔR^2^*** |
| **Step1** |  |  |  | 0.03 |  |  |  | 0.09^***^ |  |  |  | 0.08^***^ |
| Gender | 0.20 | 0.10 | 0.12 |  | 0.36 | 0.09 | 0.20^***^ |  | 0.37 | 0.09 | 0.23^***^ |  |
| Education | -0.16 | 0.09 | -0.10 |  | -0.28 | 0.08 | -0.18^***^ |  | -0.31 | 0.08 | -0.22^***^ |  |
| Marital status | -0.03 | 0.11 | -0.02 |  | 0.11 | 0.08 | 0.07 |  | -0.02 | 0.08 | -0.01 |  |
| Employment | 0.17 | 0.09 | 0.11 |  | -0.32 | 0.15 | -0.11^*^ |  | 0.09 | 0.19 | 0.03 |  |
| **Step2** |  |  |  | 0.39^***^ |  |  |  | 0.28^***^ |  |  |  | 0.28^***^ |
| Gender | 0.07 | 0.08 | 0.04 |  | 0.18 | 0.08 | 0.10^*^ |  | 0.16 | 0.08 | 0.10^*^ |  |
| Education | -0.11 | 0.07 | -0.07 |  | -0.11 | 0.07 | -0.07 |  | -0.15 | 0.07 | -0.10^*^ |  |
| Marital status | -0.08 | 0.09 | -0.04 |  | 0.07 | 0.07 | 0.04 |  | -0.06 | 0.07 | -0.04 |  |
| Employment | 0.07 | 0.07 | 0.04 |  | -0.19 | 0.12 | -0.06 |  | 0.18 | 0.16 | 0.06 |  |
| POMS tension-anxiety | -0.01 | 0.01 | -0.05 |  | -0.00 | 0.01 | -0.02 |  | 0.02 | 0.01 | 0.14 |  |
| POMS anger-hostility | -0.00 | 0.01 | -0.04 |  | -0.00 | 0.01 | -0.03 |  | -0.03 | 0.01 | -0.22^**^ |  |
| Perceived anxiety | 0.55 | 0.04 | 0.65^***^ |  | 0.50 | 0.04 | 0.57^***^ |  | 0.44 | 0.04 | 0.53^***^ |  |
| **Step 3** |  |  |  | 0.00 |  |  |  | 0.00 |  |  |  | 0.00 |
| Gender | 0.07 | 0.08 | 0.04 |  | 0.18 | 0.08 | 0.10^*^ |  | 0.15 | 0.08 | 0.09 |  |
| Education | -0.11 | 0.07 | -0.07 |  | -0.11 | 0.07 | -0.07 |  | -0.15 | 0.07 | -0.10^*^ |  |
| Marital status | -0.08 | 0.09 | -0.04 |  | 0.07 | 0.07 | 0.04 |  | -0.07 | 0.07 | -0.04 |  |
| Employment | 0.07 | 0.07 | 0.04 |  | -0.19 | 0.13 | -0.06 |  | 0.18 | 0.16 | 0.05 |  |
| POMS tension-anxiety | -0.01 | 0.01 | -0.05 |  | -0.00 | 0.01 | -0.03 |  | 0.02 | 0.01 | 0.14 |  |
| POMS anger-hostility | -0.00 | 0.01 | -0.04 |  | -0.00 | 0.01 | -0.03 |  | -0.03 | 0.01 | -0.22^**^ |  |
| Perceived anxiety | 0.55 | 0.04 | 0.65^***^ |  | 0.50 | 0.04 | 0.57^***^ |  | 0.43 | 0.04 | 0.53^***^ |  |
| Risk vulnerability | -0.01 | 0.06 | -0.01 |  | 0.00 | 0.05 | 0.00 |  | 0.02 | 0.05 | 0.02 |  |
| **Step 4** |  |  |  | 0.00 |  |  |  | 0.00 |  |  |  | 0.00 |
| Gender | 0.07 | 0.08 | 0.04 |  | 0.18 | 0.08 | 0.10^*^ |  | 0.15 | 0.08 | 0.10 |  |
| Education | -0.11 | 0.07 | -0.07 |  | -0.11 | 0.07 | -0.07 |  | -0.15 | 0.07 | -0.10 |  |
| Marital status | -0.08 | 0.09 | -0.04 |  | 0.08 | 0.07 | 0.05 |  | -0.07 | 0.07 | -0.04 |  |
| Employment | 0.07 | 0.07 | 0.04 |  | -0.18 | 0.13 | -0.06 |  | 0.17 | 0.16 | 0.05 |  |
| POMS tension-anxiety | -0.01 | 0.01 | -0.05 |  | -0.00 | 0.01 | -0.03 |  | 0.02 | 0.01 | 0.14 |  |
| POMS anger-hostility | -0.00 | 0.01 | -0.04 |  | -0.00 | 0.01 | -0.03 |  | -0.02 | 0.01 | -0.22^**^ |  |
| Perceived anxiety | 0.55 | 0.04 | 0.66^***^ |  | 0.50 | 0.04 | 0.57^***^ |  | 0.43 | 0.04 | 0.53^***^ |  |
| Risk vulnerability | -0.01 | 0.06 | -0.00 |  | -0.00 | 0.05 | -0.00 |  | 0.03 | 0.05 | 0.03 |  |
| Incidence deaths | -0.03 | 0.07 | -0.02 |  | 0.05 | 0.06 | 0.04 |  | -0.06 | 0.05 | 0.05 |  |
| **Step 5** |  |  |  | 0.01 |  |  |  | 0.00 |  |  |  | 0.00 |
| Gender | 0.05 | 0.08 | 0.03 |  | 0.18 | 0.08 | 0.10^*^ |  | 0.16 | 0.08 | 0.10^*^ |  |
| Education | -0.09 | 0.07 | -0.06 |  | -0.11 | 0.07 | -0.07 |  | -0.14 | 0.07 | -0.10 |  |
| Marital status | -0.08 | 0.09 | -0.04 |  | 0.08 | 0.07 | 0.05 |  | -0-06 | 0.07 | -0.04 |  |
| Employment | 0.06 | 0.07 | 0.04 |  | -0.18 | 0.13 | -0.06 |  | 0.16 | 0.16 | 0.05 |  |
| POMS tension-anxiety | -0.01 | 0.01 | -0.07 |  | -0.00 | 0.01 | -0.02 |  | 0.02 | 0.01 | 0.14 |  |
| POMS anger-hostility | -0.00 | 0.01 | -0.04 |  | -0.01 | 0.01 | -0.04 |  | -0.03 | 0.01 | -0.22^**^ |  |
| Perceived anxiety | 0.55 | 0.04 | 0.66^***^ |  | 0.50 | 0.05 | 0.57^***^ |  | 0.41 | 0.04 | 0.53^***^ |  |
| Risk vulnerability | -0.02 | 0.06 | -0.01 |  | -0.00 | 0.05 | -0.00 |  | 0.02 | 0.05 | 0.02 |  |
| Incidence deaths | -0.03 | 0.07 | -0.02 |  | 0.05 | 0.06 | 0.04 |  | -0.05 | 0.05 | -0.05 |  |
| Objective health | -0.07 | 0.12 | -0.03 |  | 0.08 | 0.11 | 0.03 |  | 0.00 | 0.09 | 0.00 |  |
| Subjective health | -0.08 | 0.05 | -0.90 |  | 0.05 | 0.04 | 0.05 |  | -0.06 | 0.05 | -0.06 |  |
| Note: ^***^ *p* < .001; ^**^ *p* < .01; ^*^ *p* < .05  Gender was coded 0 = male, 1 = female; Education was coded 0 = not having university degree, 1 = having university degree; Marital status was coded 0 = unmarried, 1 = married; Employment was coded 0 = not working; 1 = working. | | | | | | | | | | | | |

**Table S4.** *Hierarchical regression analysis to predict risk severity in age groups 50-59, 60-69, and over 70.*

|  | **Age group 50-59** | | | | **Age group 60-69** | | | | **Age group over 70** | | | |
| --- | --- | --- | --- | --- | --- | --- | --- | --- | --- | --- | --- | --- |
| **Step and predictor variable** | ***B*** | ***SE B*** | ***β*** | ***ΔR^2^*** | ***B*** | ***SE B*** | ***β*** | ***ΔR^2^*** | ***B*** | ***SE B*** | ***β*** | ***ΔR^2^*** |
| **Step 1** |  |  |  | 0.02 |  |  |  | 0.03* |  |  |  | 0.05 |
| Gender | 0.14 | 0.11 | 0.08 |  | 0.04 | 0.08 | 0.03 |  | 0.15 | 0.10 | 0.13 |  |
| Education | -0.10 | 0.09 | -0.06 |  | -0.15 | 0.07 | -0.11^*^ |  | -0.18 | 0.09 | -0.16 |  |
| Marital status | 0.10 | 0.09 | 0.06 |  | -0.04 | 0.08 | -0.03 |  | 0.11 | 0.11 | 0.09 |  |
| Employment | 0.22 | 0.13 | 0.10 |  | -0.12 | 0.07 | -0.10 |  | -0.02 | 0.10 | -0.02 |  |
| **Step2** |  |  |  | 0.36^***^ |  |  |  | 0.25^***^ |  |  |  | 0.15^***^ |
| Gender | -0.07 | 0.09 | -0.04 |  | -0.05 | 0.07 | -0.04 |  | 0.06 | 0.09 | 0.06 |  |
| Education | -0.02 | 0.07 | -0.01 |  | -0.09 | 0.07 | -0.07 |  | -0.12 | 0.09 | -0.11 |  |
| Marital status | 0.00 | 0.07 | 0.00 |  | -0.01 | 0.07 | -0.00 |  | 0.08 | 0.10 | 0.07 |  |
| Employment | 0.06 | 0.11 | 0.02 |  | -0.18 | 0.06 | -0.14^**^ |  | -0.03 | 0.10 | -0.02 |  |
| POMS tension-anxiety | 0.01 | 0.01 | 0.10 |  | -0.01 | 0.01 | -0.05 |  | 0.03 | 0.02 | 0.20 |  |
| POMS anger-hostility | -0.03 | 0.01 | -0.23^**^ |  | -0.01 | 0.01 | -0.0 |  | -0.03 | 0.01 | -0.25^*^ |  |
| Perceived anxiety | 0.50 | 0.04 | 0.61^***^ |  | 0.42 | 0.04 | 0.54^***^ |  | 0.25 | 0.06 | 0.35^***^ |  |
| **Step 3** |  |  |  | 0.01 |  |  |  | 0.10^*^ |  |  |  | 0.00 |
| Gender | -0.07 | 0.09 | -0.04 |  | -0.04 | 0.07 | -0.03 |  | 0.06 | 0.09 | 0.05 |  |
| Education | -0.02 | 0.07 | -0.01 |  | -0.10 | 0.06 | -0.08 |  | -0.13 | 0.09 | -0.11 |  |
| Marital status | 0.00 | 0.07 | 0.00 |  | -0.01 | 0.07 | -0.01 |  | 0.10 | 0.10 | 0.08 |  |
| Employment | 0.03 | 0.11 | 0.01 |  | -0.18 | 0.06 | -0.14^**^ |  | -0.04 | 0.10 | -0.03 |  |
| POMS tension-anxiety | 0.01 | 0.01 | 0.08 |  | -0.01 | 0.01 | -0.04 |  | 0.03 | 0.02 | 0.20 |  |
| POMS anger-hostility | -0.03 | 0.01 | -0.22^**^ |  | -0.01 | 0.01 | -0.07 |  | -0.03 | 0.01 | -0.26^*^ |  |
| Perceived anxiety | 0.48 | 0.04 | 0.59^***^ |  | 0.38 | 0.04 | 0.49^***^ |  | 0.23 | 0.06 | 0.32^***^ |  |
| Risk vulnerability | 0.09 | 0.05 | 0.09 |  | 0.10 | 0.05 | 0.11^*^ |  | 0.07 | 0.08 | 0.08 |  |
| **Step 4** |  |  |  | 0.00 |  |  |  | 0.00 |  |  |  | 0.00 |
| Gender | -0.09 | 0.09 | -0.05 |  | -0.04 | 0.07 | -0.03 |  | 0.06 | 0.09 | 0.05 |  |
| Education | -0.02 | 0.07 | -0.01 |  | -0.10 | 0.06 | -0.08 |  | -0.13 | 0.09 | -0.11 |  |
| Marital status | 0.01 | 0.07 | 0.01 |  | -0.01 | 0.07 | -0.01 |  | 0.10 | 0.10 | 0.08 |  |
| Employment | 0.03 | 0.10 | 0.01 |  | -0.18 | 0.06 | -0.14^**^ |  | -0.04 | 0.10 | -0.03 |  |
| POMS tension-anxiety | 0.01 | 0.01 | 0.07 |  | -0.00 | 0.01 | -0.04 |  | 0.03 | 0.02 | 0.20 |  |
| POMS anger-hostility | -0.03 | 0.01 | -0.21^**^ |  | -0.01 | 0.01 | -0.07 |  | -0.03 | 0.01 | -0.26^*^ |  |
| Perceived anxiety | 0.48 | 0.04 | 0.59^***^ |  | 0.38 | 0.05 | 0.49^***^ |  | 0.23 | 0.06 | 0.32^***^ |  |
| Risk vulnerability | 0.09 | 0.05 | 0.08 |  | 0.10 | 0.05 | 0.11^*^ |  | 0.07 | 0.08 | 0.08 |  |
| Incidence deaths | 0.08 | 0.05 | 0.07 |  | 0.02 | 0.05 | 0.02 |  | 0.02 | 0.07 | 0.01 |  |
| **Step 5** |  |  |  | 0.00 |  |  |  | 0.00 |  |  |  | 0.02 |
| Gender | -0.11 | 0.10 | -0.06 |  | -0.04 | 0.07 | -0.03 |  | 0.08 | 0.09 | 0.07 |  |
| Education | -0.02 | 0.07 | -0.01 |  | -0.09 | 0.07 | -0.07 |  | -0.14 | 0.09 | -0.12 |  |
| Marital status | 0.00 | 0.07 | 0.00 |  | -0.00 | 0.07 | -0.00 |  | 0.09 | 0.10 | 0.07 |  |
| Employment | 0.03 | 0.11 | 0.01 |  | -0.18 | 0.06 | -0.14^**^ |  | -0.03 | 0.10 | -0.02 |  |
| POMS tension-anxiety | 0.01 | 0.01 | 0.08 |  | -0.01 | 0.01 | -0.05 |  | 0.03 | 0.02 | 0.19 |  |
| POMS anger-hostility | -0.03 | 0.01 | -0.22^**^ |  | -0.01 | 0.01 | -0.07 |  | -0.03 | 0.01 | -0.24^*^ |  |
| Perceived anxiety | 0.48 | 0.04 | 0.59^***^ |  | 0.38 | 0.05 | 0.49^***^ |  | 0.20 | 0.06 | 0.27^**^ |  |
| Risk vulnerability | 0.08 | 0.05 | 0.08 |  | 0.09 | 0.05 | 0.10 |  | 0.08 | 0.08 | 0.09 |  |
| Incidence deaths | 0.09 | 0.05 | 0.08 |  | 0.01 | 0.05 | 0.01 |  | 0.04 | 0.07 | 0.04 |  |
| Objective health | -0.09 | 0.08 | -0.06 |  | -0.00 | 0.06 | -0.00 |  | 0.10 | 0.06 | 0.14 |  |
| Subjective health | -0.04 | 0.05 | -0.04 |  | -0.06 | 0.05 | -0.07 |  | -0.00 | 0.07 | -0.00 |  |
| Note: ^***^ *p* < .001; ^**^ *p* < .01; ^*^ *p* < .05  Gender was coded 0 = male, 1 = female; Education was coded 0 = not having university degree, 1 = having university degree; Marital status was coded 0 = unmarried, 1 = married; Employment was coded 0 = not working; 1 = working. | | | | | | | | | | | | |

**Table S5.** *Hierarchical regression analysis to predict risk vulnerability in age groups 18-29, 30-39, and 40-49.*

|  | **Age group 18-29** | | | | **Age group 30-39** | | | | **Age group 40-49** | | | |
| --- | --- | --- | --- | --- | --- | --- | --- | --- | --- | --- | --- | --- |
| **Step and predictor variable** | ***B*** | ***SE B*** | ***β*** | ***ΔR^2^*** | ***B*** | ***SE B*** | ***β*** | ***ΔR^2^*** | ***B*** | ***SE B*** | ***β*** | ***ΔR^2^*** |
| **Step 1** |  |  |  | 0.01 |  |  |  | 0.02^*^ |  |  |  | 0.04^**^ |
| Gender | -0.06 | 0.09 | -0.04 |  | 0.17 | 0.08 | 0.11^*^ |  | 0.39 | 0.10 | 0.22^***^ |  |
| Education | 0.05 | 0.08 | 0.04 |  | 0.01 | 0.07 | 0.01 |  | -0.05 | 0.09 | -0.03 |  |
| Employment | 0.11 | 0.08 | 0.08 |  | 0.32 | 0.14 | 0.12^*^ |  | 0.12 | 0.21 | 0.03 |  |
| **Step2** |  |  |  | .19^***^ |  |  |  | 0.12^***^ |  |  |  | 0.14^***^ |
| Gender | -0.08 | 0.08 | -0.05 |  | 0.08 | 0.08 | 0.05 |  | 0.24 | 0.10 | 0.14^*^ |  |
| Education | 0.05 | 0.07 | 0.04 |  | 0.10 | 0.07 | 0.07 |  | 0.06 | 0.09 | 0.04 |  |
| Employment | 0.06 | 0.07 | 0.05 |  | 0.39 | 0.13 | 0.15^**^ |  | 0.18 | 0.19 | 0.05 |  |
| POMS depression-dejection | -0.00 | 0.01 | -0.00 |  | 0.01 | 0.01 | 0.11 |  | 0.02 | 0.01 | 0.15 |  |
| POMS confusion-bewilderment | -0.00 | 0.01 | -0.03 |  | 0.01 | 0.01 | 0.04 |  | 0.02 | 0.01 | 0.09 |  |
| POMS tension-anxiety | 0.02 | 0.01 | 0.13 |  | -0.02 | 0.01 | -0.21^*^ |  | 0.00 | 0.01 | 0.03 |  |
| POMS anger-hostility | 0.01 | 0.01 | 0.14 |  | -0.00 | 0.01 | -0.00 |  | -0.00 | 0.01 | -0.02 |  |
| POMS fatigue-inertia | -0.01 | 0.01 | -0.05 |  | 0.01 | 0.01 | 0.04 |  | -0.02 | 0.02 | -0.11 |  |
| POMS vigor-activity | 0.01 | 0.01 | 0.06 |  | -0.00 | 0.01 | -0.04 |  | 0.01 | 0.01 | 0.06 |  |
| Perceived anxiety | 0.25 | 0.04 | 0.34^***^ |  | 0.26 | 0.04 | 0.34^***^ |  | 0.27 | 0.05 | 0.32^***^ |  |
| **Step 3** |  |  |  | 0.00 |  |  |  | 0.00 |  |  |  | 0.00 |
| Gender | -0.08 | 0.08 | -0.05 |  | 0.07 | 0.08 | 0.05 |  | 0.24 | 0.10 | 0.14^*^ |  |
| Education | 0.05 | 0.07 | 0.04 |  | 0.10 | 0.07 | 0.07 |  | 0.06 | 0.09 | 0.04 |  |
| Employment | 0.06 | 0.07 | 0.05 |  | 0.39 | 0.13 | 0.15^**^ |  | 0.18 | 0.19 | 0.05 |  |
| POMS depression-dejection | -0.00 | 0.01 | -0.00 |  | 0.01 | 0.01 | 0.12 |  | 0.02 | 0.01 | 0.15 |  |
| POMS confusion-bewilderment | -0.00 | 0.01 | -0.03 |  | 0.01 | 0.01 | 0.04 |  | 0.02 | 0.01 | 0.09 |  |
| POMS tension-anxiety | 0.01 | 0.01 | 0.13 |  | -0.02 | 0.01 | -0.21^*^ |  | 0.00 | 0.01 | 0.02 |  |
| POMS anger-hostility | 0.01 | 0.01 | 0.14 |  | -0.00 | 0.01 | -0.00 |  | 0.00 | 0.01 | 0.02 |  |
| POMS fatigue-inertia | -0.01 | 0.01 | -0.05 |  | 0.01 | 0.01 | 0.04 |  | -0.02 | 0.02 | -0.11 |  |
| POMS vigor-activity | 0.01 | 0.01 | 0.06 |  | -0.00 | 0.01 | -0.04 |  | 0.01 | 0.01 | 0.06 |  |
| Perceived anxiety | 0.25 | 0.05 | 0.34^***^ |  | 0.26 | 0.05 | 0.34^***^ |  | 0.26 | 0.06 | 0.31^***^ |  |
| Risk severity | -0.00 | 0.06 | -0.00 |  | 0.01 | 0.05 | 0.01 |  | 0.02 | 0.07 | 0.02 |  |
| **Step 4** |  |  |  | 0.04^**^ |  |  |  | 0.02^*^ |  |  |  | 0.05^***^ |
| Gender | -0.11 | 0.08 | -0.07 |  | 0.06 | 0.08 | 0.04 |  | 0.21 | 0.10 | 0.12* |  |
| Education | 0.05 | 0.07 | 0.03 |  | 0.08 | 0.07 | 0.06 |  | 0.01 | 0.08 | 0.01 |  |
| Employment | 0.05 | 0.07 | 0.04 |  | 0.38 | 0.13 | 0.14^**^ |  | 0.19 | 0.19 | 0.05 |  |
| POMS depression-dejection | -0.00 | 0.01 | -0.05 |  | 0.01 | 0.01 | 0.12 |  | 0.01 | 0.01 | 0.14 |  |
| POMS confusion-bewilderment | -0.01 | 0.01 | -0.04 |  | 0.00 | 0.01 | 0.03 |  | 0.01 | 0.01 | 0.07 |  |
| POMS tension-anxiety | 0.02 | 0.01 | 0.18 |  | -0.02 | 0.01 | -0.20^*^ |  | 0.00 | 0.01 | 0.02 |  |
| POMS anger-hostility | 0.01 | 0.01 | 0.10 |  | -0.00 | 0.01 | -0.01 |  | 0.00 | 0.01 | 0.02 |  |
| POMS fatigue-inertia | -0.00 | 0.01 | -0.01 |  | 0.00 | 0.01 | 0.03 |  | -0.02 | 0.02 | -0.09 |  |
| POMS vigor-activity | 0.00 | 0.01 | 0.03 |  | -0.01 | 0.01 | -0.05 |  | 0.01 | 0.01 | 0.05 |  |
| Perceived anxiety | 0.22 | 0.05 | 0.31^***^ |  | 0.25 | 0.05 | 0.33^***^ |  | 0.21 | 0.06 | 0.25^***^ |  |
| Risk severity | 0.01 | 0.06 | 0.01 |  | 0.01 | 0.05 | 0.01 |  | 0.09 | 0.07 | 0.08 |  |
| Incidence infections | 0.19 | 0.06 | 0.20^**^ |  | 0.10 | 0.05 | 0.11^*^ |  | 0.25 | 0.06 | 0.26^***^ |  |
| Incidence deaths | 0.03 | 0.07 | 0.03 |  | 0.06 | 0.06 | 0.06 |  | -0.07 | 0.07 | -0.06 |  |
| **Step 5** |  |  |  | 0.01^*^ |  |  |  | 0.00 |  |  |  | 0.01 |
| Gender | -0.12 | 0.08 | -0.08 |  | 0.06 | 0.08 | 0.04 |  | 0.22 | 0.10 | 0.13^*^ |  |
| Education | 0.06 | 0.07 | 0.05 |  | 0.08 | 0.07 | 0.06 |  | 0.02 | 0.08 | 0.01 |  |
| Employment | 0.04 | 0.07 | 0.03 |  | 0.38 | 0.13 | 0.15^**^ |  | 0.19 | 0.19 | 0.05 |  |
| POMS depression-dejection | -0.00 | 0.01 | -0.05 |  | 0.01 | 0.01 | 0.12 |  | 0.02 | 0.01 | 0.15 |  |
| POMS confusion-bewilderment | -0.01 | 0.01 | -0.04 |  | 0.00 | 0.01 | 0.03 |  | 0.01 | 0.01 | 0.05 |  |
| POMS tension-anxiety | 0.02 | 0.01 | 0.16 |  | -0.02 | 0.01 | -0.20^*^ |  | 0.00 | 0.01 | 0.03 |  |
| POMS anger-hostility | 0.01 | 0.01 | 0.12 |  | -0.00 | 0.01 | -0.01 |  | 0.00 | 0.01 | 0.01 |  |
| POMS fatigue-inertia | -0.00 | 0.01 | -0.03 |  | 0.00 | 0.01 | 0.03 |  | -0.02 | 0.02 | -0.11 |  |
| POMS vigor-activity | 0.01 | 0.01 | 0.04 |  | -0.01 | 0.01 | -0.05 |  | 0.01 | 0.01 | 0.07 |  |
| Perceived anxiety | 0.22 | 0.05 | 0.31^***^ |  | 0.25 | 0.05 | 0.33^***^ |  | 0.21 | 0.06 | 0.25^***^ |  |
| Risk severity | -0.00 | 0.06 | -0.01 |  | 0.01 | 0.05 | 0.01 |  | 0.07 | 0.07 | 0.07 |  |
| Incidence infections | 0.20 | 0.06 | 0.20^**^ |  | 0.10 | 0.05 | 0.10^*^ |  | 0.23 | 0.06 | 0.24^***^ |  |
| Incidence deaths | 0.03 | 0.07 | 0.02 |  | 0.07 | 0.06 | 0.06 |  | -0.06 | 0.07 | -0.05 |  |
| Subjective health | -0.10 | 0.04 | -0.12^*^ |  | -0.02 | 0.05 | -0.02 |  | -0.10 | 0.06 | -0.09 |  |
| Note: ^***^ *p* < .001; ^**^ *p* < .01; ^*^ *p* < .05  Gender was coded 0 = male, 1 = female; Education was coded 0 = not having university degree, 1 = having university degree; Marital status was coded 0 = unmarried, 1 = married; Employment was coded 0 = not working; 1 = working. | | | | | | | | | | | | |

**Table S6.** *Hierarchical regression analysis to predict risk vulnerability in age groups 50-59, 60-69, and over 70.*

|  | **Age group 50-59** | | | | **Age group 60-69** | | | | **Age group over 70** | | | |
| --- | --- | --- | --- | --- | --- | --- | --- | --- | --- | --- | --- | --- |
| **Step and predictor variable** | ***B*** | ***SE B*** | ***β*** | ***ΔR^2^*** | ***B*** | ***SE B*** | ***β*** | ***ΔR^2^*** | ***B*** | ***SE B*** | ***β*** | ***ΔR^2^*** |
| **Step 1** |  |  |  | 0.03^*^ |  |  |  | 0.01 |  |  |  | 0.04 |
| Gender | 0.17 | 0.10 | 0.09 |  | -0.02 | 0.08 | -0.02 |  | 0.24 | 0.10 | 0.19^*^ |  |
| Education | 0.05 | 0.08 | 0.03 |  | 0.04 | 0.08 | 0.03 |  | 0.01 | 0.10 | 0.01 |  |
| Employment | 0.31 | 0.12 | 0.14^*^ |  | 0.10 | 0.08 | 0.07 |  | 0.07 | 0.12 | 0.05 |  |
| **Step 2** |  |  |  | 0.12^***^ |  |  |  | 0.15^***^ |  |  |  | 0.20^***^ |
| Gender | 0.05 | 0.10 | 0.03 |  | -0.08 | 0.08 | -0.05 |  | 0.15 | 0.10 | 0.12 |  |
| Education | 0.10 | 0.08 | 0.06 |  | 0.08 | 0.07 | 0.06 |  | 0.08 | 0.10 | 0.06 |  |
| Employment | 0.20 | 0.12 | 0.09 |  | 0.04 | 0.07 | 0.03 |  | 0.13 | 0.11 | 0.09 |  |
| POMS depression-dejection | -0.02 | 0.01 | -0.13 |  | -0.01 | 0.01 | -0.06 |  | -0.01 | 0.02 | -0.09 |  |
| POMS confusion-bewilderment | 0.00 | 0.01 | 0.00 |  | 0.00 | 0.02 | -0.00 |  | 0.01 | 0.02 | 0.04 |  |
| POMS tension-anxiety | 0.02 | 0.01 | 0.12 |  | -0.01 | 0.02 | -0.06 |  | -0.00 | 0.02 | -0.01 |  |
| POMS anger-hostility | -0.03 | 0.01 | -0.21^*^ |  | -0.01 | 0.01 | -0.06 |  | 0.02 | 0.02 | 0.11 |  |
| POMS fatigue-inertia | 0.03 | 0.02 | 0.16 |  | 0.03 | 0.02 | 0.17 |  | 0.02 | 0.02 | 0.13 |  |
| POMS vigor-activity | -0.01 | 0.01 | -0.05 |  | 0.01 | 0.01 | 0.09 |  | 0.00 | 0.01 | 0.04 |  |
| Perceived anxiety | 0.23 | 0.04 | 0.30^***^ |  | 0.33 | 0.05 | 0.41^***^ |  | 0.32 | 0.06 | 0.40^***^ |  |
| **Step 3** |  |  |  | 0.01* |  |  |  | 0.01 |  |  |  | 0.00 |
| Gender | 0.07 | 0.10 | 0.04 |  | -0.07 | 0.08 | -0.05 |  | 0.15 | 0.10 | 0.12 |  |
| Education | 0.10 | 0.08 | 0.07 |  | 0.10 | 0.07 | 0.07 |  | 0.09 | 0.10 | 0.07 |  |
| Employment | 0.19 | 0.12 | 0.09 |  | 0.06 | 0.07 | 0.04 |  | 0.13 | 0.11 | 0.09 |  |
| POMS depression-dejection | -0.01 | 0.01 | -0.13 |  | -0.01 | 0.01 | -0.06 |  | -0.01 | 0.02 | -0.10 |  |
| POMS confusion-bewilderment | 0.00 | 0.01 | -0.01 |  | -0.00 | 0.02 | -0.01 |  | 0.01 | 0.02 | 0.05 |  |
| POMS tension-anxiety | 0.01 | 0.01 | 0.10 |  | -0.01 | 0.02 | -0.05 |  | -0.00 | 0.02 | -0.02 |  |
| POMS anger-hostility | -0.02 | 0.01 | -0.18^*^ |  | -0.01 | 0.01 | -0.05 |  | 0.02 | 0.02 | 0.13 |  |
| POMS fatigue-inertia | 0.03 | 0.02 | 0.18 |  | 0.03 | 0.02 | 0.16 |  | 0.02 | 0.02 | 0.13 |  |
| POMS vigor-activity | -0.00 | 0.01 | -0.03 |  | 0.01 | 0.01 | 0.08 |  | 0.00 | 0.01 | 0.03 |  |
| Perceived anxiety | 0.17 | 0.05 | 0.21^**^ |  | 0.28 | 0.06 | 0.35^***^ |  | 0.30 | 0.07 | 0.37^***^ |  |
| Risk severity | 0.13 | 0.06 | 0.14^*^ |  | 0.12 | 0.06 | 0.12 |  | 0.08 | 0.09 | 0.07 |  |
| **Step 4** |  |  |  | 0.03^**^ |  |  |  | 0.03^**^ |  |  |  | 0.01 |
| Gender | 0.02 | 0.10 | 0.01 |  | -0.08 | 0.08 | -0.05 |  | 0.16 | 0.10 | 0.13 |  |
| Education | 0.08 | 0.08 | 0.05 |  | 0.06 | 0.07 | 0.04 |  | 0.07 | 0.10 | 0.06 |  |
| Employment | 0.12 | 0.12 | 0.05 |  | 0.07 | 0.07 | 0.05 |  | 0.12 | 0.11 | 0.08 |  |
| POMS depression-dejection | -0.01 | 0.01 | -0.12 |  | -0.01 | 0.01 | -0.06 |  | -0.01 | 0.02 | -0.10 |  |
| POMS confusion-bewilderment | -0.00 | 0.01 | -0.01 |  | 0.00 | 0.02 | 0.00 |  | 0.01 | 0.02 | 0.04 |  |
| POMS tension-anxiety | 0.01 | 0.01 | 0.09 |  | -0.01 | 0.02 | -0.07 |  | -0.01 | 0.02 | -0.03 |  |
| POMS anger-hostility | -0.02 | 0.01 | -0.18^*^ |  | -0.00 | 0.01 | -0.01 |  | 0.02 | 0.02 | 0.13 |  |
| POMS fatigue-inertia | 0.03 | 0.01 | 0.18^*^ |  | 0.03 | 0.02 | 0.14 |  | 0.03 | 0.02 | 0.14 |  |
| POMS vigor-activity | -0.01 | 0.01 | -0.04 |  | 0.01 | 0.01 | 0.09 |  | 0.01 | 0.01 | 0.04 |  |
| Perceived anxiety | 0.17 | 0.05 | 0.21^**^ |  | 0.27 | 0.05 | 0.33^***^ |  | 0.30 | 0.07 | 0.36^***^ |  |
| Risk severity | 0.11 | 0.06 | 0.12 |  | 0.13 | 0.06 | 0.13 |  | 0.08 | 0.09 | 0.07 |  |
| Incidence infections | 0.19 | 0.06 | 0.21^***^ |  | 0.20 | 0.06 | 0.21^**^ |  | 0.10 | 0.10 | 0.10 |  |
| Incidence deaths | -0.06 | 0.07 | -0.05 |  | -0.10 | 0.07 | 0.09 |  | 0.01 | 0.11 | 0.13 |  |
| **Step 5** |  |  |  | 0.02^*^ |  |  |  | 0.00 |  |  |  | 0.01 |
| Gender | 0.02 | 0.10 | 0.01 |  | -0.07 | 0.08 | -0.05 |  | 0.17 | 0.10 | 0.13 |  |
| Education | 0.09 | 0.08 | 0.06 |  | 0.07 | 0.07 | 0.05 |  | 0.08 | 0.10 | 0.06 |  |
| Employment | 0.11 | 0.12 | 0.05 |  | 0.07 | 0.07 | 0.05 |  | 0.14 | 0.11 | 0.10 |  |
| POMS depression-dejection | -0.01 | 0.01 | -0.11 |  | -0.01 | 0.01 | -0.08 |  | -0.01 | 0.02 | -0.09 |  |
| POMS confusion-bewilderment | -0.00 | 0.01 | -0.01 |  | -0.00 | 0.02 | -0.01 |  | 0.00 | 0.02 | 0.01 |  |
| POMS tension-anxiety | 0.01 | 0.01 | 0.09 |  | -0.01 | 0.02 | -0.06 |  | -0.00 | 0.02 | -0.02 |  |
| POMS anger-hostility | -0.02 | 0.01 | -0.17^*^ |  | -0.00 | 0.01 | -0.01 |  | 0.01 | 0.02 | 0.14 |  |
| POMS fatigue-inertia | 0.03 | 0.01 | 0.15 |  | 0.02 | 0.02 | 0.14 |  | 0.03 | 0.02 | 0.14 |  |
| POMS vigor-activity | 0.00 | 0.01 | -0.00 |  | 0.01 | 0.01 | 0.09 |  | 0.01 | 0.01 | 0.08 |  |
| Perceived anxiety | 0.15 | 0.05 | 0.20^**^ |  | 0.27 | 0.05 | 0.33^***^ |  | 0.28 | 0.07 | 0.35^***^ |  |
| Risk severity | 0.11 | 0.06 | 0.11 |  | 0.13 | 0.06 | 0.12 |  | 0.07 | 0.09 | 0.06 |  |
| Incidence infections | 0.19 | 0.06 | 0.21^***^ |  | 0.20 | 0.06 | 0.21^**^ |  | 0.10 | 0.10 | 0.10 |  |
| Incidence deaths | -0.05 | 0.07 | -0.05 |  | -0.10 | 0.07 | -0.09 |  | 0.03 | 0.11 | 0.03 |  |
| Subjective health | -0.12 | 0.05 | -0.13^*^ |  | -0.05 | 0.05 | -0.06 |  | -0.09 | 0.07 | -0.10 |  |
| Note: ^***^ *p* < .001; ^**^ *p* < .01; ^*^ *p* < .05  Gender was coded 0 = male, 1 = female; Education was coded 0 = not having university degree, 1 = having university degree; Marital status was coded 0 = unmarried, 1 = married; Employment was coded 0 = not working; 1 = working. | | | | | | | | | | | | |
